# Does stress consistently favor habits over goal-directed behaviors? Data from two preregistered exact replication studies

**DOI:** 10.1016/j.ynstr.2023.100528

**Published:** 2023-02-20

**Authors:** Tom Smeets, Stephanie M. Ashton, Simone J.A.A. Roelands, Conny W.E.M. Quaedflieg

**Affiliations:** a Department of Medical and Clinical Psychology, Center of Research on Psychological disorders and Somatic diseases (CoRPS), Tilburg University, the Netherlands; b Department of Neuropsychology & Neuropharmacology, Maastricht University, the Netherlands

**Keywords:** Stress, Cortisol, Instrumental learning, Goal-directed behavior, Habits

## Abstract

Instrumental learning is controlled by two distinct parallel systems: goal-directed (action-outcome) and habitual (stimulus-response) processes. Seminal research by Schwabe and Wolf (2009, 2010) has demonstrated that stress renders behavior more habitual by decreasing goal-directed control. More recent studies yielded equivocal evidence for a stress-induced shift towards habitual responding, yet these studies used different paradigms to evaluate instrumental learning or used different stressors. Here, we performed exact replications of the original studies by exposing participants to an acute stressor either before (cf. Schwabe and Wolf, 2009) or directly after (cf. Schwabe and Wolf, 2010) an instrumental learning phase in which they had learned that distinct actions led to distinct, rewarding food outcomes (i.e., instrumental learning). Then, following an outcome devaluation phase in which one of the food outcomes was consumed until participants were satiated, action-outcome associations were tested in extinction. Despite successful instrumental learning and outcome devaluation and increased subjective and physiological stress levels following stress exposure, the stress and no-stress groups in both replication studies responded indifferently to valued and devalued outcomes. That is, non-stressed participants failed to demonstrate goal-directed behavioral control, thereby rendering the critical test of a shift from goal-directed to habitual control in the stress group inapt. Several reasons for these replication failures are discussed, including the rather indiscriminate devaluation of outcomes that may have contributed to indifferent responding during extinction, which emphasize the need to further our understanding of the boundary conditions in research aimed at demonstrating a stress-induced shift towards habitual control.

## 1 Introduction

Human behavior is shaped by learning that specific actions lead to specific outcomes, which helps us to adapt and respond to our ever-changing environment. The performance of instrumental actions is controlled by a goal-directed and habit system (O'Doherty et al., 2017; Wood and Rünger, 2016; de Wit and Dickinson, 2009). Initially, instrumental actions are largely goal-directed, driven by rewards and the causal relation between stimulus and response. However, with extended training, those actions become increasingly more automatic (i.e., habitual; see de Wit and Dickinson, 2009, but see de Wit et al., 2018 and Pool et al., 2022 for recent discussions on how overtraining affects the formation of habit-like behaviors). Thus, certain stimuli trigger automatic responses regardless of the value of that stimulus (Balleine and O'Doherty, 2010). While such habitual behavior is more efficient, it often comes at the cost of behavioral flexibility (de Wit and Dickinson, 2009; Tricomi, Balleine, & O'Doherty, 2009). The inability to flexibly update stimulus-outcome associations – which promotes adaptive behavior – is associated with psychopathology such as obsessive-compulsive disorder (Gillan et al., 2011; Gillan et al., 2014; van der Straten et al., 2020), eating disorders (Voon et al., 2014) or drug addiction (Schwabe et al., 2011).

Ground-breaking work by Schwabe and Wolf (2009, 2010) revealed that, in humans, acute stress shifts the balance from goal-directed to habitual processes. Using Valentin, Dickinson, and O'Doherty (2007)'s selective satiation paradigm, Schwabe and Wolf (2009) demonstrated that participants exposed to acute stress *before* instrumental learning were insensitive to outcome devaluation, and subsequently responded more habitual than non-stressed controls. In a follow-up study, Schwabe and Wolf (2010) found that also stress *after* instrumental learning and outcome devaluation led to more habitual responses during extinction. Since the publication of these two seminal studies and a close replication using the same outcome devaluation paradigm (Schwabe et al., 2011a, Schwabe et al., 2011b), the finding that stress promotes the expression of habitual behavior has been demonstrated in studies that used similar outcome devaluation paradigms (e.g., Hartogsveld et al., 2020; Pritchard et al., 2018; for review see Schwabe and Wolf, 2013; Wirz et al., 2018), studies employing probabilistic classification learning tasks (e.g., Schwabe and Wolf, 2012; Schwabe, Tegenthoff, Hö;ffken, & Wolf, 2013) and research using sequential decision-making tasks measuring model-based versus model-free learning (which are indicative of goal-directed vs. habitual learning, respectively; Meier et al., 2021; Otto et al., 2013; Radenbach et al., 2015). Moreover, several studies demonstrated that the activation of major stress response systems (i.e., the sympatho-adrenal-medullary and hypothalamic-pituitary-adrenal axes) may stimulate habitual control over goal-directed control (e.g., Schwabe et al., 2010; 2011). Notably, a shift from goal-directed to habitual control over behavior is deemed adaptive in times of stress since habitual control is less cognitively demanding than goal-directed control (for review see Schwabe and Wolf, 2013; Wirz et al., 2018).

However, the above-mentioned conceptual replications on how acute stress may foster habitual behavior have not been unequivocal and several boundary conditions have by now been identified. In studies that used a sequential decision-making task, for example, a stress-induced shift from goal-directed to habitual behavior was only found in those participants who presented with low working memory (Otto et al., 2013) or when acute stress was combined with chronic stress effects (Radenbach et al., 2015). Likewise, studies using an outcome devaluation paradigm other than the one used in the original work by Schwabe and Wolf (2009, 2010), either found no effect of stress applied before instrumental learning on the fostering of habits (Buabang et al., 2022; Fournier, d'Arripe-Longueville and Radel, 2017), or found a shift towards habits only for participants displaying strong cortisol reactions to the acute stressor (Smeets et al., 2019) or for participants with low baseline working memory (Quaedflieg et al., 2019). In summary, how and under which specific conditions acute stress may induce a shift from goal-directed to habitual behavior remains an open question.

The current work constitutes a direct replication of the Schwabe and Wolf (2009, 2010) studies using the original outcome devaluation paradigm and following the same procedures to further evaluate the robustness of their findings. Participants first engage in an instrumental learning task in which they learn that two specific actions lead to two distinct rewarding outcomes. Subsequently, participants are selectively devalued for one of the food outcomes by eating that food to satiety. Finally, the previously learned instrumental actions are tested in extinction. A decrease in the frequency of the action associated with the devalued outcome is indicative of flexible goal-directed behavior, whereas a continued preference for the devalued outcome reflects more rigid, habitual, behavior. Crucially, participants are exposed to an acute stressor either before the instrumental learning phase (Study 1; cf. Schwabe and Wolf, 2009) or after instrumental learning and selective outcome devaluation (Study 2; cf. Schwabe and Wolf, 2010). Given the exact nature of these replications, we hypothesized that stress would prompt habitual behavior at the expense of goal-directed actions in both studies. That is, as per Schwabe and Wolf (2009, 2010), we expect participants exposed to acute stress to choose the devalued high probability action significantly more often when comparing the last block of instrumental learning and the first extinction block when compared to non-stressed control participants.

## 2 Methods

### 2.1 Preregistration

All study protocols were preregistered on the Open Science Framework and can be found here: https://osf.io/ygctz and https://osf.io/5j239.

### 2.2 Determination of sample size

The *a-priori* power calculation with G* Power (α = 0.05, 1-β = 0.95, as Cohen) for the Schwabe and Wolf (2009) study, was based on the reported F-statistics (F_(1,56)_ = 13.7, *p* < .001) for the critical 3-way interaction with Condition (2 groups) × Stimulus type (2 values) x Phase (last learning versus first extinction block). The resulting η_p_^2^ of 0.196 indicated a total sample size of *N* = 72. The *a-priori* power calculation with G* Power (α = 0.05, 1-β = 0.95, as Cohen) for the Schwabe and Wolf (2010) study was based on the effect size (η_p_^2^ = 0.23), reported for the 4-way interaction of Group (2 groups) × Sex (2 groups) × Stimulus type (2 values) x Phase (last learning versus first extinction block) interaction, and yielded a total required sample size of *N* = 84. Based on the original studies we expected a drop-out rate of 16–25% due to disliking the liquids (pleasantness below 10) or choosing the high probability less than 20% of the time. As such, we recruited 25% more participants than indicated by the power analysis, rounded off to the next number that is divisible by 4 (4 groups). Thus, with a projected *N* = 92 (46 stress [23 male; 23 female]; 46 no-stress control [23 male; 23 female]) for Study 1 (i.e., 2009 replication) and *N* = 108 (54 stress [27 male; 27 female]; 54 no-stress control [27 male; 27 female]) for Study 2 (2010 replication), our replication studies included considerably larger samples than the original Schwabe and Wolf (2009, 2010) studies.

### 2.3 Participants

As per Schwabe and Wolf (2009, 2010), participants were aged between 18 and 35 (Study 1: *M*_*age*_ = 23.55, *S.E.* = 0.36; Study 2: *M*_*age*_ = 22.07, *S.E.* = 0.40), with a BMI between 18 and 28 (Study 1: *M*_*BMI*_ = 22.18, *S.E.* = 0.23; Study 2: *M*_*BMI*_ = 22.07, *S.E.* = 0.23). Research protocols were approved by the ethics committees of the Tilburg School of Social and Behavioral Sciences at Tilburg University (#RP304) and the Faculty of Psychology and Neuroscience at Maastricht University (ERCPN-OZL_226_103_08_2020). All participants provided written informed consent and were reimbursed with university credits or a small monetary reward.

### 2.4 A priori exclusion criteria

The following exclusion criteria were evaluated via an online screening questionnaire: current or chronic diseases, diagnosed psychopathology in the last 3 years, food intolerance, current or planned diet, drug abuse (i.e., >2 times a month), alcohol drinking (i.e., >10 units per week), smoking (i.e., >10 per week), or use of any prescription medication. Females taking hormonal contraceptives were excluded from participation; free cycling females were tested in the luteal phase of their menstruation cycle as cortisol stress responses then are similar to those of men (Kirschbaum et al., 1999). Furthermore, participants were pre-screened to ensure that they found the presented foods pleasant (i.e., ratings for orange juice and chocolate milk ≥70 and oranges and chocolate pudding ≥50 on 100-point rating scales).

### 2.5 Stress induction

Participants were exposed to either the stress or a control version of the Socially Evaluated Cold Pressor Test (SECPT; Schwabe, Haddad & Schachinger, 2008). In the stress condition, participants immersed their right hand up to and including the wrist for a maximum of 3 min in ice-cold water (2 °C). During hand immersion, an experimenter monitored them, who provided negative feedback during the task and were video recorded. Participants in the control condition immersed their right hand up to and including the wrist for 3 min in lukewarm water (35–37 °C). They were not monitored by the experimenter, nor videotaped.

### 2.6 Instrumental learning task

#### 2.6.1 Phase 1: Instrumental learning phase

**Fig. 1:**
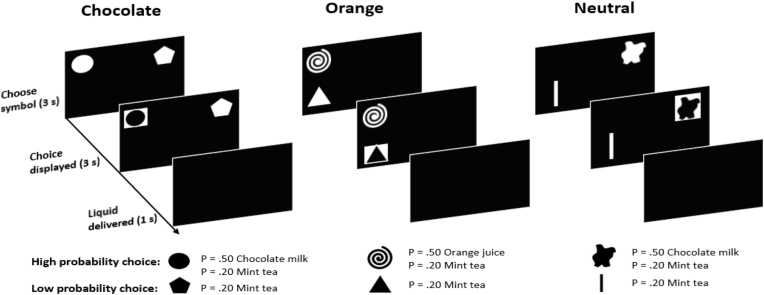
Overview of the task.

In each trial, participants had to choose one of two distinct symbols displayed on the pc screen by clicking with the mouse on the symbol of their choice (see Fig. 1). The selected symbol was highlighted for 3 s, and thereafter either 1 ml of one of the liquids was delivered or no liquid at all was delivered (depending on chosen action and outcome probabilities). Liquids were delivered with independent electronic pumps for each liquid and transferred via tubes with a diameter of 3 mm to the participants, who kept the ends of the tubes like straws between their lips.

Depending on the trial type (chocolate, orange, or neutral; with each trial type being represented by a unique set of symbols throughout the instrumental learning task), one action led to a food outcome with a probability of *p* = .7 (high probability action) whereas the other action resulted in a food outcome with a probability of *p* = .2 (low probability action). Specifically, in chocolate and orange trials, the high probability action was followed by receiving chocolate milk or orange juice, respectively, with a probability of *p* = .5, and by mint tea with a probability of *p* = .2. In neutral trials, non-sparkling water was delivered either with a probability of *p* = .7 (high probability action) or *p* = .2 (low probability action). Participants completed 75 trials in each of the three trial types (225 trials in total), presented in random order. With a trial duration of 8 s, the total length was approximately 30 min.

#### 2.6.2 Phase 2: Outcome devaluation

In the selective outcome devaluation, participants were presented with either chocolate pudding or oranges and were instructed as follows: “*eat as much as possible until you are really full, you will need to eat for at least* 5 min*”*. The amount of food consumed was comparable in the stress and control groups (all *p*'s > 0.40) and equivalent to the original studies (Study 1: Oranges: control *M* = 3.95 (*S.E.* = 0.55); stress: *M* = 3.84 (S.E. = 0.33), Chocolate cups: control *M* = 1.92 (S.E. = 0.16); stress: *M* = 2.14 (S.E. = 0.21); Study 2: Oranges: control *M* = 2.97 (S.E. = 0.27); stress: *M* = 2.78 (S.E. = 0.28), Chocolate cups: control *M* = 2.32 (S.E. = 0.21); stress: *M* = 2.40 (S.E. = 0.20)). The assignment to one of the two food devaluation conditions was counterbalanced across groups.

#### 2.6.3 Phase 3: Extinction

As in the learning stage, participants were presented with 75 trials of each of the three trial types in random order and participants again had to select symbols that previously led to different food outcomes. In this stage, however, the rewards (chocolate milk and orange juice) were not delivered (i.e., the task was performed in extinction). Instead, both actions led to the common outcome (mint tea) with a probability of *p* = .2 on reward trials. For neutral trials, water was delivered with a probability of *p* = .2 for both actions. A decrease in the selection of the devalued outcome is considered indicative of goal-directed behavior, whereas still choosing the devalued outcome is thought to reflect habitual behavior. The Instrumental Learning Task (ILT) was presented via the Psychtoolbox-3 for MATLAB.

#### 2.6.4 Explicit knowledge

At the end of the experiment, participants' explicit knowledge of the learned action-outcome associations was assessed. In Study 1 (*2009 Replication*) a recall test was used in which participants were asked to describe which symbol had to be selected to receive chocolate milk, orange juice, or water in the instrumental learning phase. Each mentioned outcome was scored as correct or incorrect, with cumulative scores for each participant having a maximum score of 3 points. In Study 2 (*2010 Replication*), there first was a recall test about the actions necessary to receive chocolate milk, orange juice, or water in the instrumental learning phase. Participants received one point for each correctly named symbol and symbol position. Secondly, a multiple-choice recognition test determined participants’ knowledge of i) the actions necessary to receive chocolate milk, orange juice, or water, and ii) the position of the six symbols. The recall test had a maximum of 6 points and the recognition test had a maximum of 9 points. Note that the explicit knowledge measure is neither a sensitive measure nor has it been found to be consistently affected by stress in the original studies (Schwabe and Wolf, 2009, 2010).

### 2.7 Measures

#### 2.7.1 Blood pressure

Systolic, diastolic blood pressure, and heart rate were measured at 4 time points: before, twice during, and immediately after the stress induction/control task. The two measures taken during the stress induction/control task were averaged to form a single value for the t_stress_ time point (see the procedure for exact timings). All measures were taken from the left arm using an Omron M7 (HEM-759-E; Omron Healthcare Europe BV).

#### 2.7.2 Cortisol

As per Schwabe and Wolf (2009, 2010), salivary cortisol was sampled using synthetic Salivettes (Sarstedt, Etten- Leur, The Netherlands) at 4 (Study 1) or 5 time points (Study 2). The samples were stored at −20 °C immediately upon collection until cortisol concentrations were determined by Dresden LabService GmbH using a commercially available chemiluminescence immunoassay (IBL Intl., Hamburg, Germany), with mean intra- and inter-assay coefficients of variation <8%.

#### 2.7.3 Subjective ratings

Subjective stress was assessed via 3 visual analogue scales (VASs; anchors 0 = “not at all”; 100 = “extremely”) on which participants rated how stressful, painful, and unpleasant the SECPT or control task had been to them.

### 2.8 Design

To avoid recruitment issues, which were especially prominent during the COVID-19 pandemic phase, Study 1 was conducted at Maastricht University while Study 2 was conducted at Tilburg University. Test sessions in both studies took approximately 2 h per participant. To avoid fluctuations in the circadian rhythm of cortisol and mimicking the 2009 and 2010 procedures by Schwabe and Wolf, testing took place between 13:00 and 17:30 (Maastricht University) or 13:00 and 17:00 (Tilburg University). In addition, participants were not allowed to eat for the 3 h before participation or do (heavy) exercise and withhold from caffeine in the last 6 h before testing.

### 2.9 Procedure

#### 2.9.1 Study 1

Participants received information regarding the test day and thereafter provided informed consent. Per the current recommendations (Shields, 2020; Strahler et al., 2017), they then completed filler questionnaires for 10 min to accommodate to the test environment. Thereafter, a baseline measure of blood pressure and cortisol (t_pre-stress_) was taken followed by participants being subjected to either the stress or no-stress version of the SECPT. Blood pressure (t_stress_) was measured twice during the SECPT (30 s after task onset and 30 s after the first measurement). Immediately after the SECPT, blood pressure and cortisol were assessed (t_post-stress_), followed by subjective ratings. Participants then were given neutral reading materials (national geographic, slower, digital SLR photography) that they could browse through during a 20-min wait period, after which a final cortisol measurement was taken (t_+20_). Next, participants completed the instrumental learning phase, with hunger and pleasantness ratings for the presented liquids measured before and after the learning phase, and finally, an ultimate saliva sample was collected (t_+50_). Subsequently, the outcome devaluation phase commenced with participants having to eat either oranges or chocolate pudding to satiation. After satiation was reached, hunger and pleasantness ratings were reassessed, after which participants completed the extinction phase. Finally, after the explicit knowledge test, participants were debriefed and renumerated.

#### 2.9.2 Study 2

**Fig. 2:**
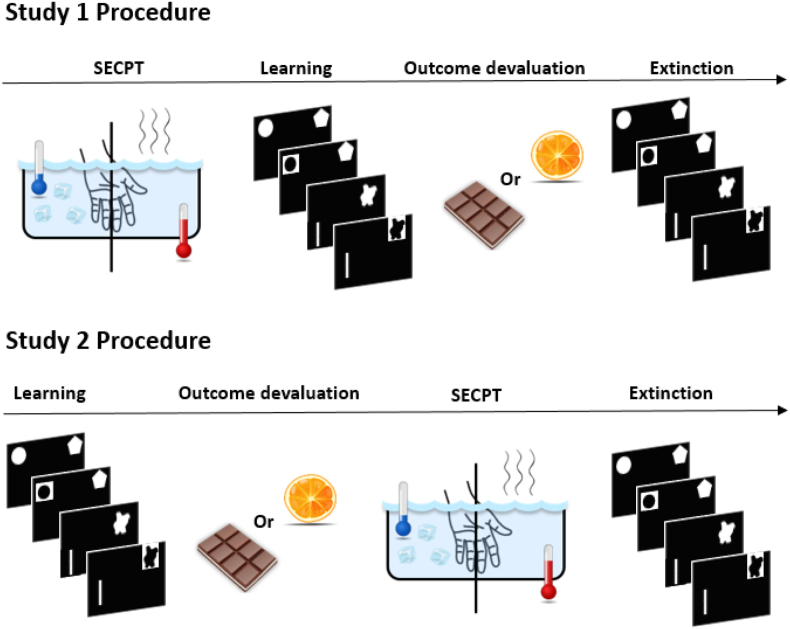
Overview of the study procedures.

Participants received information regarding the test day, provided informed consent, and then completed filler questionnaires for 10 min (cf. supra) after which baseline cortisol (t_baseline_) was assessed. Next, participants completed the instrumental learning phase, with hunger and pleasantness ratings for the presented liquids measured before and afterward. Participants then ate either oranges or chocolate pudding to satiation (i.e., outcome devaluation phase), after which hunger and pleasantness ratings were reassessed. Next, blood pressure and cortisol were assessed before (t_pre-stress_) and immediately after (t_post-stress_) participants were exposed to the SECPT, with further blood pressure measurements taken twice during the SECPT (t_stress_; cf. Study 1) and subjective stress ratings assessed once after the SECPT. Subsequently, participants browsed through neutral reading materials that were provided to them for 20 min, after which cortisol was measured (t_+20_). Participants then completed the extinction task, with hunger and pleasantness ratings assessed before the start and after completion of the extinction phase. Finally, after an explicit knowledge test and a final cortisol measure (t_+50_), participants were debriefed and renumerated (see Fig. 2).

### 2.10 Post-hoc exclusion criteria

As per the original studies, in the current studies participants with data that matches one or more of the following criteria were excluded from analysis: *(i)* participants with scores <20% on high probability trials during the instrumental learning phase; *(ii)* participants with pleasantness ratings <10 on 0–100 scale on chocolate milk or orange juice after the instrumental learning phase; and *(iii)* participants who did not fully complete all phases of the ILT.

Based on these criteria, 12 participants were excluded from Study 1 and 13 from Study 2. This resulted in a final sample of 80 participants (40 stress [21 male; 19 female]; 40 no-stress control [20 male; 20 female]) for Study 1, and a final sample of 95 (48 stress [22 male; 26 female]; 47 no-stress controls [24 male; 23female]) for Study 2.

### 2.11 Statistical analyses

In line with the original Schwabe and Wolf (2009, 2010) studies, we first checked for the specific effect of food devaluation on pleasantness ratings of the offered drinks by conducting a 3 (WS; Time: t_pre-ILT_, t_pre-devaluation_, t_post-devaluation_) x 2 (WS; Value: valued vs. devalued food) x 2 (BS; Condition: stress vs. control) repeated-measures ANOVA.

The *main analyses* focusing on whether stress leads to a preference for habitual actions used a 2 (WS; Time: last block instrumental learning vs. first block extinction) x 2 (WS; Value: valued vs. devalued food) x 2 (BS; Condition: stress vs. control) repeated-measures ANOVA, with 15 trials per block in the instrumental learning and extinction phase. This analysis was supplemented with a Bayesian repeated-measures ANOVA with Time (last block instrumental learning vs. first block extinction) and Value (valued vs. devalued food) as within-subject factors and Condition (stress vs. control) as between-subject factor.

For both studies the following additional analyses were done: *(i)* the effectiveness of the stress induction was evaluated by analyzing between-condition changes in systolic and diastolic blood pressure as well as heart rate by conducting 3 (WS; Time: t_pre-stress_, t_stress,_ t_post-stress_) x 2 (BS; Condition: stress vs. control) repeated-measures ANOVAs. Subjective stress was determined using the three VAS scales and analysed using an independent samples (stress vs. control) *t*-test; *(ii)* to check whether participants had learned the associations to gain the high probability rewards, a 5 (WS; Time: block 1, 2, 3, 4, 5) x 2 (WS; Value: valued vs. devalued) x 2 (BS; Condition: stress vs. control) repeated-measures ANOVA was conducted; and *(iii)* to check the effectiveness of the devaluation procedure on self-reported satiety, a 3 (WS; Time: t_pre-ILT_, t_pre-devaluation_, t_post-devaluation_) x2 (BS; Condition: stress vs. control) repeated-measures ANOVA was conducted.

Next, for Study 1 only, potential differences between conditions *(i)* in cortisol concentrations were determined using a 4 (WS; Time: t_baseline_, t_+1,_ t_+20,_ t_+50_) x 2 (BS; Condition: stress vs. control) repeated-measures ANOVA, *(ii)* in explicit knowledge of the learned action-outcome associations was examined using a χ^2^ test; and *(iii)* in the number of correctly recalled associations was evaluated with an independent samples *t*-test.

Also, for Study 2 only, potential differences between conditions *(i)* in cortisol concentrations were examined with a 5 (Time: t_baseline_, t_pre-stress,_ t_+1,_ t_+20,_ t_+50_) x 2 (Condition: stress vs. control) repeated-measures ANOVA; and *(ii)* in explicit knowledge of the learned action-outcome associations were evaluated via two independent sample *t*-tests on the multiple-choice explicit knowledge task (recall and recognition).

## 3 Results

### 3.1 Results: study 1

#### 3.1.1 Successful stress induction via the SECPT

Thirteen participants (8 male, 5 female) in the stress group did not keep their hand in the water for the full 3 min of the SECPT (range 35–172 s). Those who did not complete the duration had higher cortisol concentrations at baseline (*t*_(14.39)_ = −3.19, *p* = .003, *d* = 1.08) and t_+01_ (*t*_(14.59)_ = −2.50, *p* = .025, *d* = 1.05) compared to those who finished the procedure. These participants did not differ in subjective stress, blood pressure, or cortisol at t_+20_ and t_+50_ (all *t*'s < 1.26, *p*'s > 0.21, *d*'s < 0.42), and as such were included in all further analyses.

#### 3.1.2 Subjective stress ratings

The stress version of the SECPT was subjectively more stressful than the no-stress control version (*F*_(1,78)_ = 230.76, *p* < .001, η_p_^2^ = 0.75; see Table 1). Specifically, participants in the stress group found the SECPT significantly more stressful (*F*_(1,76)_ = 103.27, *p* < .001, η_p_^2^ = 0.58), painful (*F*_(1,76)_ = 327.04, *p* < .001, η_p_^2^ = 0.79) and unpleasant (*F*_(1,86)_ = 129.11, *p* < .001, η_p_^2^ = 0.60) compared to the control task for no-stress controls. Note that there was a significant interaction between condition and sex for the stress scale (*F*_(1,86)_ = 10.33, *p* = .002, η_p_^2^ = 0.11), with follow-up *t*-tests demonstrating that females rated the SECPT as more stressful than males (stress: *t*_(38)_ = 2.66, *p* = .011, *d* = 0.82). Sex did not influence ratings of unpleasantness or pain (all *F's* < 2.11, *p's* > 0.15*,* η_p_^2^ < 0.027).

**Table 1:** Means (±S.E.) of blood pressure and subjective stress for stress and No-stress controls in study 1 and study 2.

	STUDY 1						STUDY 2					
Blood	*Stress*			*No-Stress Controls*			*Stress*			*No-Stress Controls*		
Pressure	Pre-stress	Stress	Post-stress	Pre-stress	Stress	Post-stress	Pre-stress	Stress	Post-stress	Pre-stress	Stress	Post-stress
***Systolic***	117.25 (2.11)	135.80 (2.92)	117.08 (2.69)	120.18 (1.91)	114.58 (1.86)	113.25 (1.53)	121.35 (1.67)	139.79 (2.10)	120.31 (2.12)	119.62 (1.93)	116.66 (1.89)	116.70 (1.70)
***Diastolic***	75.10 (1.25)	94.30 (2.52)	78.33 (1.80)	77.13 (1.43)	75.78 (1.34)	73.43 (1.08)	73.29 (1.40)	92.15 (1.71)	75.13 (1.61)	72.43 (1.18)	72.53 (1.25)	116.70 (1.70)
**Subjective Stress**	**Stress**	**Pain**	**Un-pleasant**	**Stress**	**Pain**	**Un-pleasant**	**Stress**	**Pain**	**Un-pleasant**	**Stress**	**Pain**	**Un-pleasant**
	48.32 (4.93)	68.30 (4.01)	75.10 (4.05)	1.33 (0.58)	1.35 (0.57)	17.82 (3.70)	45.48 (3.77)	75.77 (2.35)	77.00 (3.62)	3.40 (0.82)	3.32 (0.90)	20.00 (3.49)

#### 3.1.3 Blood pressure

Participants in the stress group had significantly higher blood pressure following the SECPT compared to no-stress controls (SBP: *F*_(2,152)_ = 52.79, *p* < .001, η_p_^2^ = 0.41; DBP: *F*_(1.8, 136.80)_ = 42.33, *p* < .001, η_p_^2^ = 0.36; see Table 1), with no significant differences between sexes (main and interactive effects: all *F*'s < 2.55, *p*'s > 0.082, η_p_^2^ < 0.032). Follow-up tests per time point revealed a significantly higher BP for the stress group relative to the no-stress control group during the SECPT for systolic and diastolic blood pressure (SBP: *F*_(1,78)_ = 37.57, *p* < .001, η_p_^2^ = 0.33; DBP: *F*_(1,78)_ = 42.10, *p* < .001, η_p_^2^ = 0.35) and at the post-stress measure for diastolic blood pressure only (*F*_(1,78)_ = 5.47, *p* = .022, η_p_^2^ = 0.066). No significant differences were observed at baseline (both *F*'s < 1.13, *p*'s > 0.29, η_p_^2^'s = 0.014) or post-stress for systolic blood pressure (*F*_(1,78)_ = 1.13, *p* = .29, η_p_^2^ = 0.014).

#### 3.1.4 Salivary cortisol

**Fig. 3:**
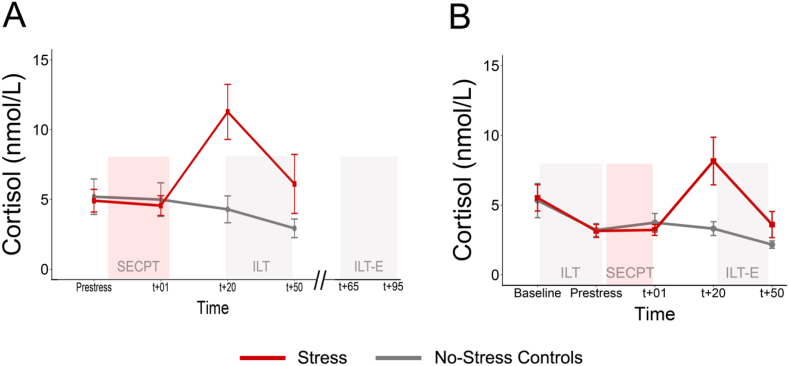
Successful stress induction in both studies. Mean raw cortisol levels and 95% confidence intervals for the stress and no-stress control condition in Study 1 (panel A) and Study 2 (panel B).

Two participants in the control condition had missing data for the baseline sample and were excluded from these specific analyses. Participants in the stress group had significantly higher cortisol concentrations following the SECPT (Time x Condition: *F*_(1.59, 117.93)_ = 23.41, *p* < .001, η_p_^2^ = 0.24, see Fig. 3). Follow-up tests revealed a significant difference between the conditions at t_+20_ and t_+50_ (t_+20_: *F*_(1,78)_ = 41.35, *p* < .001, η_p_^2^ = 0.35; t_+50_: *F*_(1,78)_ = 8.42, *p* = .005, η_p_^2^ = 0.097). No significant differences were observed at baseline or at t_+01_ (both *F*'s < 0.39, *p*'s > 0.53, η_p_^2^ < 0.005). These results were not found to differ between sexes (*F*'s < 0.97, *p*'s > 0.37, η_p_^2^'s < 0.013).

#### 3.1.5 Effects of stress on instrumental learning

Participants who showed no increase in the choice of the high probability action during the learning phase (i.e., choosing <20% of chocolate or orange trials) were excluded from the analysis. Of the 11 excluded, 1 stress and 1 no-stress control participant could not name any of the action-outcome associations for the three trial types.

**Fig. 4:**
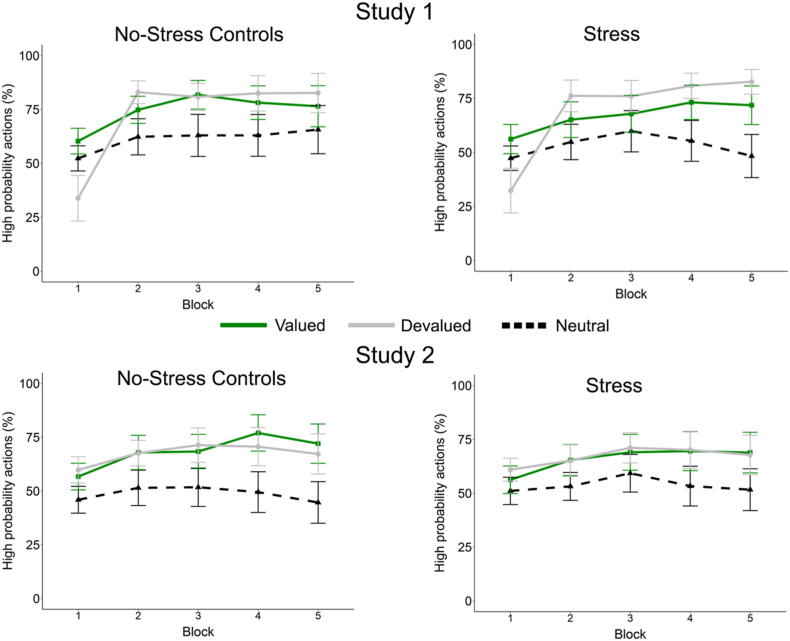
Mean percentage and 95% confidence intervals of high probability choices across blocks in the instrumental learning task for the stress and no-stress control groups for both studies.

Replicating the original findings, participants learned to choose the high probability action more often than the low probability action (i.e., neutral trials; see Fig. 4). The mixed ANOVA model showed a main effect of Value (*F*_(1.55, 117.89)_ = 18.91, *p* < .001, η_p_^2^ = 0.20) and Time (*F*_(2.72, 206.70)_ = 61.56, *p* < .001, η_p_^2^ = 0.47**)** and a significant Time × Value interaction (*F*_(4.85, 368.24)_ = 24.90, *p* < .001, η_p_^2^ = 0.25). Importantly, there was no effect of stress on learning (Time x Condition x Value: *F*_(4.85, 368.24)_ = 1.60, *p* = .16, η_p_^2^ = 0.021). In addition, Sex did not influence the results (all *F*'s < 2.17 *p*'s > 0.099, η_p_^2^ < 0.028). Follow-up analyses for the fifth and last learning block demonstrated that there was a significant main effect of Value, with Bonferroni-corrected comparisons showing that the percentage high probability actions for devalued outcomes was higher than for valued outcomes (*p* = .016), which in turn was higher than for neutral outcomes (*p* = .001).

#### 3.1.6 Effects of outcome devaluation on subjective hunger and pleasantness ratings

The devaluation procedure significantly reduced hunger ratings (*F*_(1,76)_ = 315.06, *p* < .001, η_p_^2^ = 0.81). Hunger ratings decreased from 57.24 (*SE* = 2.49) before devaluation to 8.47 (*SE* = 1.54) after devaluation. In addition, pleasantness ratings reduced following devaluation (Time x Value: *F*_(1,76)_ = 12.92, *p* = .001, η_p_^2^ = 0.15). These ratings were not influenced by Condition or Sex (all *F*'s < 2.47 *p*'s > 0.12, η_p_^2^ < 0.031). Follow up tests showed a main effect of time, with pleasantness ratings reducing for both the devalued (*F*_(1,76)_ = 31.29, *p* < .001, η_p_^2^ = 0.29) and valued (*F*_(1,76)_ = 9.56, *p* = .003, η_p_^2^ = 0.11) foods. No significant difference was observed between the devalued and valued foods before the extinction test (*t*_(80)_ = −1.21, *p* = .230, *d* = 0.14, see Table 2), implying that the devaluation procedure was unselective.

**Table 2:** Means (± S.E.) of Hunger ratings for Stress and No-Stress Controls in Study 1 and Study 2.

	HUNGER					
	*Stress*			*No-Stress Controls*		
**Study 1**	**Pre-deval**	**Post-deval**		**Pre-deval**	**Post-deval**	
	57.95 (3.50)	8.03 (2.15)		56.37 (2.15)	8.68 (2.15)	
**Study 2**	**Pre-deval**	**Post-deval**	**Pre-ext**	**Pre-deval**	**Post-deval**	**Pre-ext**
	61.97	16.82	28.55	50.03	7.50	15.82
	(2.99)	(2.56)	(3.20)	(3.01)	(2.57)	(3.22)

Abbreviations: deval = devaluation, ext = extinction.

#### 3.1.7 Effects of outcome devaluation and stress on instrumental responses in the extinction test

**Fig. 5:**
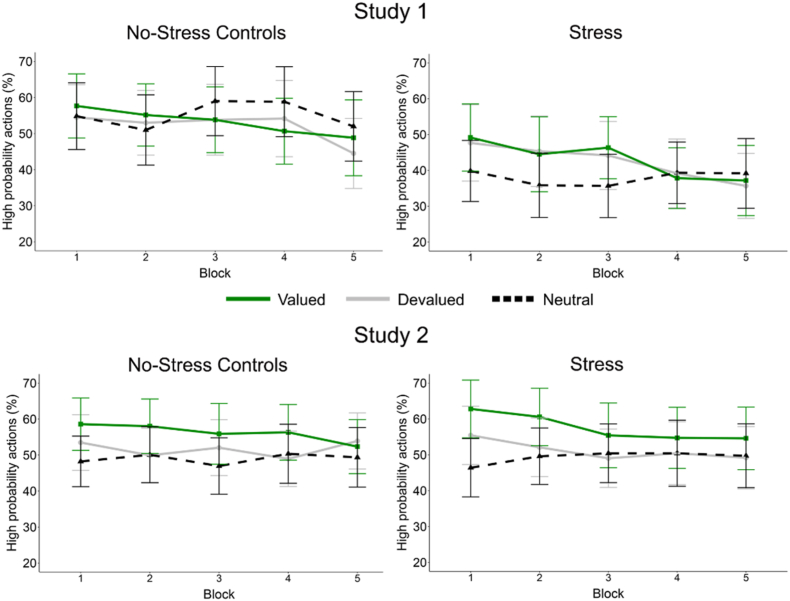
Mean percentage and 95% confidence intervals of high probability choices across the extinction test for the stress and no-stress control groups for both studies.

Habitual and goal-directed action in the extinction task were analysed with a mixed ANOVA with Condition (stress vs. no stress controls) x Sex (male vs. female) x Value (valued vs. devalued) x Time (5 blocks of the extinction task). A significant main effect of Time was observed (*F*_(3.04, 230.59)_ = 5.72, *p* < .001, η_p_^2^ = 0.07), however this was not found to differ between Condition, Value or Sex (all *F*'s < 0.87, *p*'s > 0.48, η_p_^2^'s < 0.011, see Fig. 5). Stress and no stress control participants thus did not demonstrate differential responding to the valued and devalued outcomes across extinction trials.

**Fig. 6:**
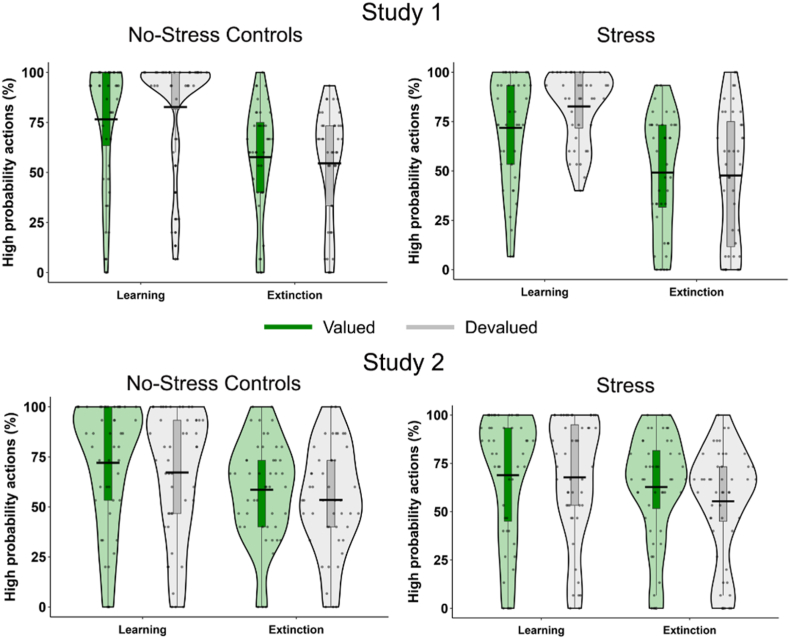
Comparison of valued and devalued high probability choices in the last 15-trial **training block and the first 15-trial testing block after outcome devaluation for the stress and control groups for both studies.** Violin plots display the distribution of the data, group means (indicated by the black bar) and individual data points.

In line with the analysis of the original paper, we also observed choice action in the last block of the learning task versus the first block of the extinction task (see Fig. 6). Results showed a main effect of Time (*F*_(1, 76)_ = 54.01, *p* < .001, η_p_^2^ = 0.45) and a Time × Value interaction (*F*_(1, 76)_ = 5.56, *p* = .021, η_p_^2^ = 0.068), demonstrating decreased responding to both the valued and devalued high probability action between time points. This was not influenced by Condition or Sex (all *F*'s < 3.25, *p*'s > 0.076, η_p_^2^'s < 0.041). Supporting Bayesian analysis indicated strong evidence against a 3-way interaction (BF_excl_ = 37.00), or main effect of stress (BF_excl_ = 4.64).

#### 3.1.8 Explicit knowledge

Stress was not found to impair participants' awareness of the action-outcome associations. Eighty percent of the stress group and 77.5% of the no-stress control group got all the explicit knowledge correct (χ^2^_(1)_ = 0.64, *p* = .80). No significant difference was observed between conditions (*t*_(78)_ = −0.252, *p* = .802, *d* = 0.056; stress: *M* = 2.6, *SE* = 0.13; no-stress controls: *M* = 2.55, *SE* = 0.15). Scores on the explicit knowledge tests were not found to correlate with the first or third block of the extinction task (both *r*'s < 0.031, *p*'s > 0.78).

### 3.2 Results: study 2

#### 3.2.1 Instrumental learning

Throughout the instrumental learning task, participants increasingly selected the high probability actions for the rewarding food types (chocolate milk and orange juice; see Fig. 4). The mixed ANOVA with Condition (stress vs. no stress controls) x Sex (male vs. female) x Value (valued, devalued, neutral) x Time (5 blocks of the learning task) showed a main effect of Time (*F*_(2.47, 224.39)_ = 8.34, *p* < .001, η_p_^2^ = 0.084), Value (*F*_(1.80, 163.56)_ = 26.41, *p* < .001, η_p_^2^ = 0.23) and Time × Value interaction (*F*_(5.61, 510.22)_ = 2.87, *p* = .004, η_p_^2^ = 0.031). All other main effects and interactions were non-significant (all *F*'s < 1.68, *p*'s > 0.19, η_p_^2^ < 0.018). Follow-up analyses for the fifth and last learning block demonstrated that there was a significant main effect of Value, with Bonferroni-corrected comparisons showing that the percentage high probability actions for neutral outcomes was significantly lower than for both valued and devalued outcomes (both *p*'s < .001), which did not differ from each other (*p* > .99).

#### 3.2.2 Effects of outcome devaluation on subjective hunger and pleasantness ratings

Subjective ratings of hunger reduced significantly after eating the devalued food to satiety (*F*_(1.66, 150.74)_ = 233.99, *p* < .001, η_p_^2^ = 0.72, see Table 2). Ratings reduced from 60.00 (*SE* = 2.12) after learning to 12.16 (*SE* = 1.81) after devaluation and 22.18 (*SE* = 2.28) before extinction. Hunger ratings did not differ between Condition or Sex (all *F*'s < 1.63, *p*'s > 0.20, η_p_^2^ < 0.018).

Pleasantness ratings for the presented foods (see Table 3) changed over the course of the experiment (Time x Value: *F*_(3.72, 334.33)_ = 12.69, *p* < .001, η_p_^2^ = 0.12). Follow-up tests showed that pleasantness ratings significantly decreased after devaluation for the devalued food (*p* < .001) and the valued food (i.e., the food not eaten; *p* = .002). The pleasantness of the devalued food was lower than of the valued food after devaluation (*t*_(94)_ = −4.74, *p* < .001, *d* = 0.49) and was still lower before the critical extinction test (*t*_(94)_ = −4.79, *p* < .001, *d* = 0.49). Pleasantness ratings did not change significantly for the neutral trials (*p* > .99). These ratings did not differ between Condition or Sex (all *F*'s < 2.32, *p*'s > 0.062, η_p_^2^ < 0.025).

**Table 3:** Means (±S.E.) of Pleasantness ratings for Stress and No-Stress Controls in Study 1 and Study 2.

	**PLEASANTNESS**																							
	***Stress***												***No-Stress Controls***											
	**Valued**				**Devalued**				**Common**				**Valued**				**Devalued**				**Common**			
**Study 1**	**Pre-deval**		**Post-deval**		**Pre-deval**		**Post-deval**		**Pre-deval**		**Post-deval**		**Pre-deval**		**Post-deval**		**Pre-deval**		**Post-deval**		**Pre-deval**		**Post-deval**	
	81.70		78.48		86.38		76.03		51.30		50.83		84.11		81.78		85.39		79.05		58.64		59.35	
	(2.58)		(2.61)		(1.79)		(2.66)		(4.12)		(4.17)		(2.59)		(2.61)		(1.80)		(2.67)		(4.13)		(4.18)	
**Study 2**	**Pre-learn**	**Post-learn**	**Post-deval**	**Pre-ext**	**Pre-learn**	**Post-learn**	**Post-deval**	**Pre-ext**	**Pre-learn**	**Post-learn**	**Post-deval**	**Pre-ext**	**Pre-learn**	**Post-learn**	**Post-deval**	**Pre-ext**	**Pre-learn**	**Post-learn**	**Post-deval**	**Pre-ext**	**Pre-learn**	**Post-learn**	**Post-deval**	**Pre-ext**
	81.08	79.66	73.92	75.17	81.62	80.23	73.92	75.17	64.30	51.98	54.55	53.98	82.54	83.37	78.85	78.54	83.07	75.67	69.57	69.52	61.67	48.13	48.39	48.22
	(1.92)	(1.92)	(1.92)	(1.92)	(1.88)	(2.85)	(1.92)	(1.92)	(3.97)	(3.95)	(4.08)	(4.12)	(1.95)	(2.38)	(2.85)	(2.95)	(1.91)	(2.90)	(3.60)	(3.54)	(4.05)	(4.03)	(4.16)	(4.20)

#### 3.2.3 Successful stress induction via the SECPT

Sixteen participants (5 male, 11 female) in the stress group did not keep their hand in the water for the full 3 min of the SECPT (range 22–165 s^1^). Those who did not complete the full SECPT reported higher subjective pain (*t*_(45.22)_ = −3.45, *p* = .001, *d* = 0.88) and lower diastolic blood pressure at the post-stress measure (*t*_(46)_ = 2.29, *p* = .027, *d* = 0.70) compared to those who finished the procedure. These participants did not differ in other subjective stress (i.e. stressfulness and unpleasantness), blood pressure, or the cortisol measures (all *p*'s > 0.23), and as such were included in all further analyses.

#### 3.2.4 Subjective stress ratings

Participants in the stress condition reported the SECPT to be significantly more stressful (*F*_(1,91)_ = 114.65, *p* < . 001, η_p_^2^ = 0.56), painful (*F*_(1,91)_ = 832.74, *p* < .001, η_p_^2^ = 0.90) and unpleasant (*F*_(1,91)_ = 124.71, *p* < .001, η_p_^2^ = 0.58) compared to no-stress controls. These results did not differ as a result of Sex (all *F*'s < 3.076, *p*'s > 0.083, η_p_^2^ < 0.0.033, see Table 1).

#### 3.2.5 Blood pressure

Participants in the stress group had significantly higher blood pressure following the SECPT compared to no-stress controls (SBP: *F*_(2,182)_ = 70.58, *p* < .001, η_p_^2^ = 0.44; DBP: *F*_(2, 182)_ = 54.05, *p* < .001, η_p_^2^ = 0.37). These results did not differ as a result of Sex (all *F*'s < 1.06, *p*'s > 0.36, η_p_^2^ < 0.0.01). Follow-up tests revealed a significant difference between the Conditions during the SECPT (SBP: *F*_(1,93)_ = 66.58, *p* < .001, η_p_^2^ = 0.42; DBP: *F*_(1,93)_ = 85.50, *p* < .001, η_p_^2^ = 0.48) and at the post-stress measure for diastolic blood pressure (*F*_(1,93)_ = 5.34, *p* = .023, η_p_^2^ = 0.054). No significant differences were observed at baseline (both *F*'s < 0.46, *p*'s > 0.50, η_p_^2^ < 0.005) or post-stress for systolic blood pressure (*F*_(1,93)_ = 1.76, *p* = .19, η_p_^2^ = 0.019; see Table 2).

#### 3.2.6 Salivary cortisol

Participants in the stress group had significantly higher cortisol concentrations following the SECPT (Condition x Time: *F*_(2.13, 191.61)_ = 17.57, *p* < .001, η_p_^2^ = 0.16; see Fig. 3). These results did not differ between sexes (both *F*'s < 1.28, *p*'s > 0.28 η_p_^2^'s < 0.014). Follow-up *t*-tests revealed a significant difference between conditions at t_+20_ (*t*_(54.83)_ = −5.48, *p* < .001, *d* = 1.12) and t_+50_ (*t*_(53.19)_ = −2.97, *p* = .004, *d* = 0.61). No significant differences were observed at baseline or immediately after (t_+01)_ the SECPT (both *F*'s < 1.91, *p*'s > 0.17, η_p_^2^ < 0.020).

#### 3.2.7 Effects of outcome devaluation and stress on instrumental responses in the extinction test

Habitual and goal-directed action in the extinction task were analysed with a mixed ANOVA with Condition (stress vs. no stress controls) x Sex (male vs. female) x Value (valued, devalued, neutral) x Time (5 blocks of the extinction task). There was a main effect of Value (*F*_(2, 182)_ = 4.56, *p* = .012, η_p_^2^ = 0.048) with valued high probability symbol chosen more than neutral ((*p*_bonferroni corrected_ = .01) while the devalued high probability symbol was not chosen more than the valued (*p*_bonferroni corrected_ = .11) or neutral (*p*_bonferroni corrected_>.99) ones. There was a marginally significant change over time (Time x Value: *F*_(6.2, 564.19)_ = 2.06, *p* = .054, η_p_^2^ = 0.022). The outcomes of the extinction task were not influenced by Condition or Sex (all *F*'s < 1.91, *p*'s > 0.17, η_p_^2^ < 0.020; See Fig. 5), meaning that stress and no stress control participants did not differ in response pattern.

In line with the analysis of the original paper, we also observed choice action in the last block of the learning task versus the first block of the extinction task (see Fig. 6). We observed a significant 4-way Time x Value x Condition × Sex interaction (*F*_(1, 91)_ = 5.21, *p* = .025, η_p_^2^ = 0.054). Follow-up tests per condition revealed significant main effects of time in the stress group (*F*_(1, 46)_ = 7.13, *p* = .010, η_p_^2^ = 0.13) and no-stress control group (*F*_(1, 45)_ = 12.15, *p* = .001, η_p_^2^ = 0.21), with both groups showing a reduction in the choice of the high probability action between the two time points. In addition, we observed a significant Time × Sex interaction in the stress group (*F*_(1, 46)_ = 6.63, *p* = .013, η_p_^2^ = 0.13). All other main effects and interactions were non-significant (all *F*'s < 1.91, *p*'s > 0.17, η_p_^2^ < 0.020). Supporting Bayesian analysis indicated strong evidence against a 3-way interaction (BF_excl_ = 155.50), or main effect of stress (BF_excl_ = 7.68).

#### 3.2.8 Explicit knowledge

At the end of the extinction task, participants were tested on their explicit knowledge for action-outcome associations (6 points possible^2^) and, in addition, stimulus-outcome associations and stimulus positions (9 points possible). The average score for the action-outcome associations was 4.21 (*SE* = 0.21) and the average for stimulus-outcome associations and stimulus positions was 7.92 (*SE* = 0.16). Both explicit knowledge tests were not influenced by Condition or Sex (all *F*'s < 2.25, *p*'s > 0.10, η_p_^2^ < 0.0.024).

## 4 Discussion

In two direct replication experiments, we aimed to evaluate the robustness of the stress-induced shift to habitual behavior control. Based on Schwabe and Wolf (2009, 2010) we expected that, compared to non-stressed controls, participants who were exposed to acute stress would choose the devalued high probability action more often when comparing the last block of instrumental learning and the first extinction block. The instrumental learning in the current replication studies was successful, as shown by an increase in selecting the high probability symbol and demonstrating strong explicit knowledge of the learned associations. Notably, the instrumental learning and explicit knowledge levels in the current studies were similar to the original studies and were not affected by stress. In contrast to our hypotheses, the results of both experiments failed to provide evidence that acute stress prompts habitual behavior, with additional Bayesian analyses providing substantial evidence for the null hypotheses. By showing that stress does not unequivocally result in habit-dominated behaviors, the current results align well with a recent conceptual replication using taste aversion as devaluation demonstrating that people under stress remain sensitive to outcome values and thus continue to prefer goal-directed behaviors (Buabang et al., 2022). Moreover, our findings also accord with previous conceptual replications that used different paradigms and in which a stress-induced shift to habitual behavior control was only found under certain boundary conditions, such as low working memory capacity (Otto et al., 2013; Quaedflieg et al., 2019), chronic stress (Radenbach et al., 2015), or strong cortisol reactions to the acute stressor (Smeets et al., 2019). Of course, there may be various reasons why our studies, in contrast to the original Schwabe and Wolf (2009, 2010) studies, did not find evidence for habitual control over behavior following acute stress exposure relative to non-stress controls. We will discuss several of those reasons in the next paragraphs.

It is worth noting first that, as close replications can be challenging (Stroebe and Strack, 2014), we contacted the lead author of the original studies (LS) multiple times to obtain all original materials and ask for clarification whenever needed and that, thankfully, the lead author was very responsive in helping us create identical conditions as per the original studies. Thus, both experiments were designed to match the procedure of the original studies as closely as possible to replicate the stress timing and procedure, the instructions given to participants, and the instrumental learning paradigm (including its outcome devaluation procedure). A premeditated difference with the original studies is that we substantially increased the sample size compared to the original papers to minimize the likelihood of null findings resulting from insufficient statistical power. Nonetheless, we acknowledge that despite these efforts, other and more subtle differences between the original and our replication studies (e.g., the used chocolate pudding in the outcome devaluation phase was not of the same brand but matched closely in terms of nutritional value and subjective taste) existed.

A second potential explanation for our findings has to do with the selective devaluation procedure. While the amount of food eaten during the selective outcome devaluation phase in the current studies was comparable to the original studies, the outcome devaluation procedure nevertheless resulted in distinctive patterns of changed hunger and pleasantness ratings. Specifically, the reduction in hunger was larger while the decrease in pleasantness ratings for the outcome liquids related to the devalued food was less apparent than in the original Schwabe and Wolf's (2009, 2010) papers. The logic behind *selective* outcome devaluation is that only one of the two outcomes decreases in hedonic value and decreased willingness to consume that particular outcome afterward. Continued responding to the still valuable outcome whilst simultaneously demonstrating less frequent responding to the devalued outcome than before, would be indicative of goal-directed control. Strikingly, however, data from our studies suggest that, potentially moderated by strong overall decreases in hunger, the devaluation may not have been selective enough and may have contributed to indifference in responding during the extinction test.

Indeed, data from our studies show that all participants, independent of being exposed to stress, were rather indifferent to the outcomes throughout the extinction test. To be precise, whereas in the original studies the non-stressed participants continued to choose the still valuable outcome but responded less (than during learning) to the devalued option (a pattern indicating continued goal-directed responding), non-stressed control participants in both our studies no longer displayed a preference for any of the outcomes and thus no longer displayed the expected goal-directed control. For the stressed participants on the other hand, the original studies showed a continued responding to the still valuable and the devalued outcomes (indicative of habitual responding), yet in both of our replication studies stressed participants were also indifferent to the still valuable and devalued outcomes. While the fact that non-stressed participants unexpectedly were no longer goal-directed can also be seen as a non-replication of the original finding, it is important to note that this also implies that a valid test of the stress-induced shift from goal-directed to habitual control, was no longer possible.

An alternative interpretation of the current results could be that the satiation manipulation affected only incentive motivation or ‘wanting’ for the food and did not affect the ‘liking’ or hedonic impact of the outcome liquids (see Berridge et al. (2009) for the difference between wanting, liking and learning). The fact that the participants liked the liquids is not surprising as, in accordance with the original studies, participants were pre-screened to ensure that they liked the liquids used as outcomes (i.e., ratings ≥70 on scale of 0–100). Though, the original papers excluded 20.2% of the participants because they disliked at least one of the liquids (i.e., ratings ≤10 on scale of 100) after the learning phase. In the current studies, only 3% of participants needed to be excluded based due to a pleasantness rating below 10 on the test day itself. Such sample differences might partly explain these divergent findings, yet this would nonetheless question the robustness of the original stress-induced habitual behavior effect. Furthermore, it is worth mentioning that only 20–25% of the screened participants could be included in the current studies based on the original in-and exclusion criteria, which demonstrates the selectiveness of this paradigm and arguably limiting the generalizability of its outcomes.

It is worth noting that it has been conjectured that the neural system for habitual control and the that for goal-directed control act in parallel to allow behavioral flexibility (Daw et al., 2005; Daw et al., 2011; O'Doherty et al., 2017; Schreiner et al., 2020). A shift can be achieved either by one form of control diminishing in influence, the other increasing, or both. This makes responding according to the learned associations between stimulus and response more likely, but not certain (Schreiner et al., 2020). This explains why, in devaluation procedures, participants still show both habitual (i.e. old) responses and goal-directed (i.e. new) responses and might provide a potential reason why shifts in goal-directed behavior have been difficult to observe in human laboratory experiments (e.g., 5 failed experiments: de Wit et al., 2018). Outcome-devaluation paradigms only probe the expression of response-outcome associations. In addition, habitual behavior in these paradigms is defined as the null hypothesis within a condition, i.e. that the outcome devaluation has no effect. This is impossible to test using frequentist statistics (De Houwer et al., 2018). As habits are defined as stimulus-response associations, these paradigms do not demonstrate the reliance on a habit per se but are said to assess gradients of goal-directed action control (Foerde, 2018; Watson and de Wit, 2018). Moreover, context effects seem to play a role in outcome-devaluation paradigms (Watson and de Wit, 2018). Studies in animals have suggested that behavior that is insensitive to outcome devaluation is highly context-dependent and subtle changes in context after outcome devaluation can bring goal-directed control back “online” via enhanced attention (Bouton, 2021; Thrailkill and Bouton, 2015). Stress has been suggested to be such context cue (Bouton, 2021) and this might explain the findings from study 2, as stress was induced after outcome devaluation.

We also acknowledge that while our samples were comparable to those of the original studies in various respects (e.g., in terms of age, applied in- and exclusion criteria, amount of food eaten during devaluation, learning rates of the instrumental learning task, etc.), one obvious difference is that our samples were recruited amidst the COVID-19 pandemic. It has been suggested that the stress associated with having to deal with this global pandemic and the restrictions imposed upon people (including wearing face masks, limiting social contact, to even complete and prolonged lockdowns) may yield unexpected effects on research conducted during the COVID-19 pandemic (e.g., Goldfarb, 2020). In our replication studies, we however found no baseline differences between the stress and control groups in stress levels (i.e., cortisol and blood pressure) that could help explain our failure to replicate the original findings. Moreover, the stress reactivity observed in the current work nicely parallels the stress reactivity observed in the original studies and the stress reactivity that is seen in previous studies that employed the SECPT in undergraduate samples (e.g., Schwabe et al., 2008; Smeets, 2011; Smeets et al., 2012).

In conclusion, by conducting exact replications of the Schwabe and Wolf (2009, 2010) studies, we aimed to assess the robustness of the acute stress-induced shift to habitual behavior. We failed to replicate the original pattern of results, as in both studies the stress and no-stress groups responded indifferently to valued and devalued outcomes, implying that non-stressed participants did not show goal-directed control and that a test of shifting from goal-directed to habitual control in the stress group was inadequate. Our failure to replicate the findings from the original studies when using experimental procedures that matched the original ones as closely as possible, albeit with larger samples sizes to increase statistical power, along with previous studies that identified several boundary conditions (e.g., Buabang et al., 2022; Quaedflieg et al., 2019; Otto et al., 2013; Smeets et al., 2019), suggests that this effect may not be particularly robust in experimental lab studies, which may in part be due to the unpredictability of the selective outcome devaluation of the instrumental learning task. Unravelling if and how acute stress affects instrumental learning is important since both stress (Furnari et al., 2015; Piazza and Le Moal, 1998; Sinha, 2001; Schwabe et al., 2011; Zorrilla et al., 2014) and an imbalance in expression of the two systems’ values, have been suggested to play a role in disorders of compulsion such as substance abuse and overeating (Everitt and Robbins, 2016; Hogarth, 2020; Vandaele and Janak, 2018). Rather than stress causing a shift from goal-directed to habitual behavior control, it is possible that stress leads to a shift in the priorities of long-term and short-term goals (Moors et al., 2017; Buabang et al., 2022). For example, stress-induced eating may be directed towards the short-term goal of reducing negative affect, rendering the response itself rewarding, raising its priority over a long-term goal of weight loss. Future studies could also employ methods like ecological momentary assessments to determine whether in real-life stress induces an overreliance on habits, and whether such excessive use of habits is critically involved in various forms of psychopathology.

## Funding

This work was supported by the Dutch Research Council (10.13039/501100003246Nederlandse Organisatie voor Wetenschappelijk Onderzoek, NWO) to prof. dr. Tom Smeets [grant number 401-19-029]. The Dutch Research Council had no further role in the study design; in the collection, analysis, and interpretation of the data; in the writing of the report; and in the decision to submit the paper for publication.

## CRediT authorship contribution statement

**Tom Smeets:** Conceptualization, Methodology, Software, Validation, Resources, Data curation, Writing – original draft, Writing – review & editing, Supervision, Project administration, Funding acquisition. **Stephanie M. Ashton:** Formal analysis, Investigation, Data curation, Writing – original draft, Writing – review & editing, Visualization. **Simone J.A.A. Roelands:** Formal analysis, Investigation, Data curation, Writing – review & editing. **Conny W.E.M. Quaedflieg:** Conceptualization, Methodology, Validation, Formal analysis, Resources, Data curation, Writing – original draft, Writing – review & editing, Supervision, Project administration.

## Declaration of competing interest

The authors declare no conflicts of interest.

## Data Availability

Data will be made available on request.

## Footnotes

1 Two participants who did not complete the full SECPT have missing data for the duration of their hand immersion.

2 The first 23 participants had a maximum score of 3 for the explicit knowledge recall. The results do not change if these participants are excluded from the sample.
